# Eyes in Staurozoa (Cnidaria): a review

**DOI:** 10.7717/peerj.6693

**Published:** 2019-04-01

**Authors:** Lucília Souza Miranda, Allen Gilbert Collins

**Affiliations:** 1 Department of Zoology, Instituto de Ciências Biológicas, Universidade Federal de Minas Gerais, Belo Horizonte, Minas Gerais, Brazil; 2 National Systematics Laboratory, National Marine Fisheries Service (NMFS), National Museum of Natural History, Smithsonian Institution, District of Columbia, WA, United States of America

**Keywords:** Stauromedusae, Ocelli, Dark pigment spot, Rhopalia, Anchors, Rhopalioids, Manania

## Abstract

The presence of dark pigment spots associated with primary tentacles (or structures derived from them, i.e., rhopalioids) in Staurozoa was recently overlooked in a study on the evolution of cnidarian eyes (defined as a “region made of photoreceptor cells adjacent to pigment cells”, irrespective of image formation, i.e., including all photoreceptive organs). Review of old and recent literature on Staurozoa shows that dark pigment spots are present in virtually all species of *Manania*, as well as some species of *Haliclystus*, *Stylocoronella*, and probably *Calvadosia*. The known ultrastructure of ocelli seems to be compatible with light perception, but no immediate response to changes in light intensity have been observed in the behavior of staurozoans. Therefore, although further studies addressing photic behavior are required, we discuss an earlier hypothesis that the dark spots in some stauromedusae may be related to synchronous spawning, as well as the possible sensorial function of rhopalioids. Observations summarized here suggest a possible ninth independent origin of eyes in Cnidaria, within a lineage of benthic medusae. Alternatively, documented similarity across medusae of Cubozoa, Scyphozoa, and Staurozoa—with eyes being topologically associated with primary tentacles in each of these taxa—could indicate shared ancestry and a single origin of eyes in this clade known as Acraspeda. Information on Staurozoa, one of the least studied groups within Cnidaria, is often neglected in the literature, but correctly recognizing the characters of this class is crucial for understanding cnidarian evolution.

## Introduction

**Figure 1:**
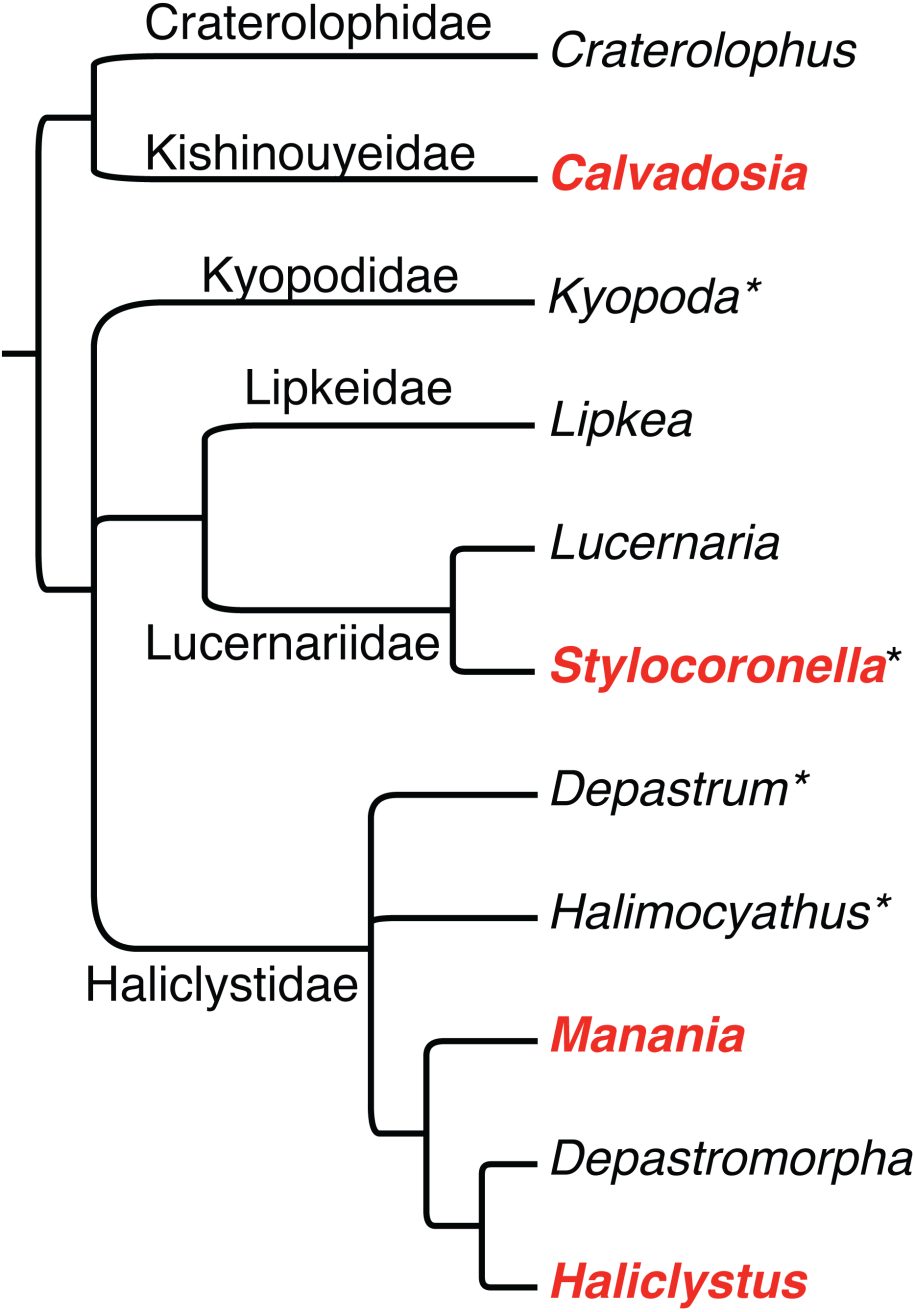
Phylogenetic hypothesis of relationships among staurozoan genera and families, based on Miranda et al. (2016a). Asterisks after generic names indicate that the genus has not yet been sampled for molecular data, and thus their respective positions in the phylogeny are based solely on inferences based on morphology. Bolded genera in red have been documented to have pigment spots, observations that are reviewed herein.

Staurozoa is a cnidarian class currently represented by 50 species classified in 11 genera (Miranda et al., 2016a; Miranda et al., 2018; Fig. 1). They are all marine, benthic, and generally reported in shallow temperate waters (Miranda et al., 2018). Staurozoa have a life cycle with two main generations (i.e., metagenetic), known as the stauropolyp and the stauromedusa (Wietrzykowski, 1912; Kikinger & Salvini-Plawen, 1995; Miranda, Collins & Marques, 2010). However, metamorphosis in Staurozoa is not so clearly defined as in other medusozoans (i.e., Cubozoa, Hydrozoa, and Scyphozoa). In Staurozoa, metamorphosis is mainly observed in the apical region (i.e., calyx) and the stauromedusa remains attached to the substrate by a basal peduncle (Wietrzykowski, 1912; Kikinger & Salvini-Plawen, 1995; Miranda, Collins & Marques, 2010). Therefore, the medusa stage has polypoid and medusoid characters (Miranda et al., 2016a).

**Figure 2:**
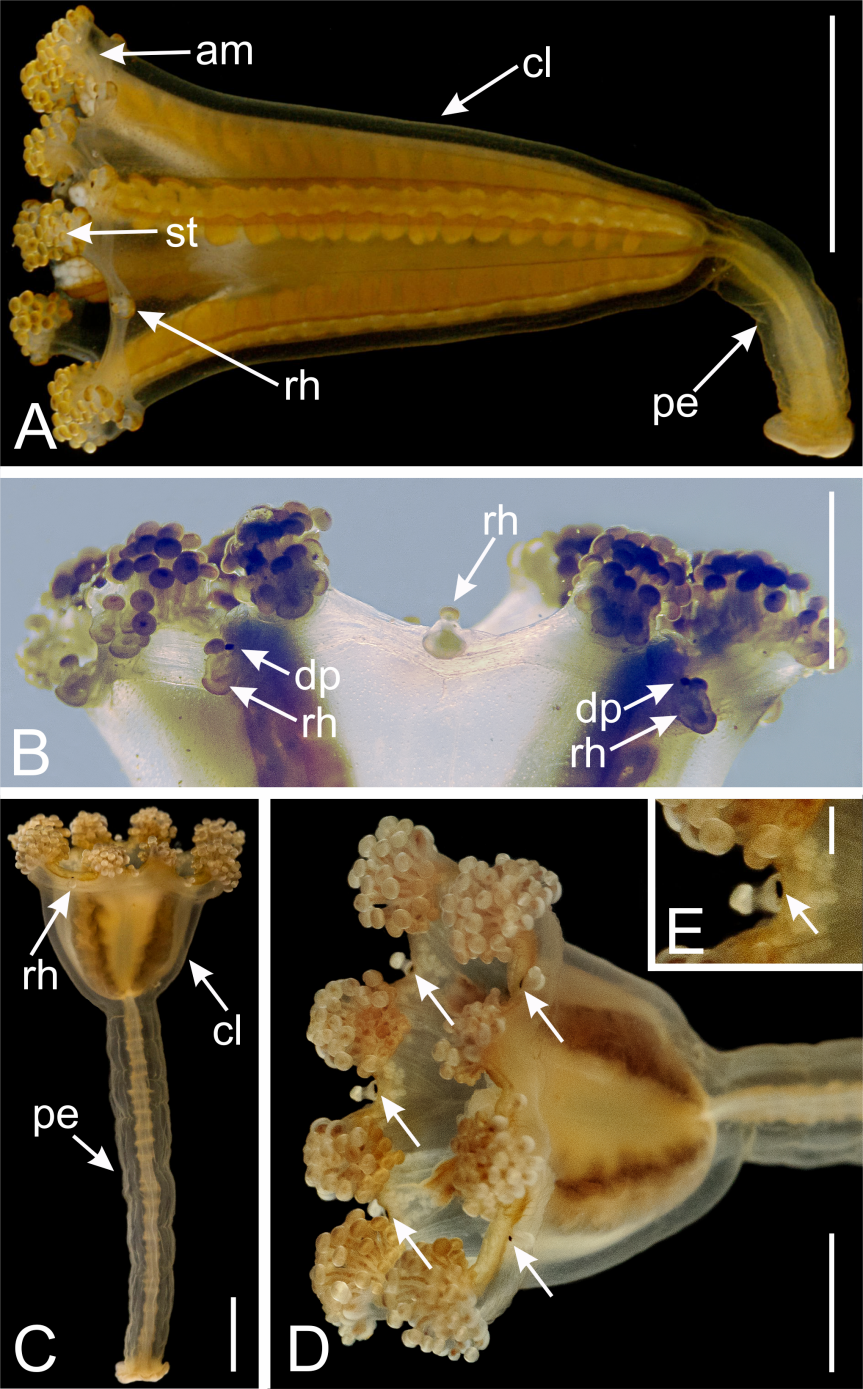
Dark pigment spots associated with rhopalioids in species of *Manania*. (A) General view of *Manania uchidai*. (B) View of calyx margin of *M. uchidai*, with rhopalioids and dark pigment spots associated with rhopalioids. (C) General view of *Manania auricula*. (D) View of calyx of *M. auricula* with arrows pointing to dark pigment spots associated with rhopalioids. (E) Arrow pointing to dark pigment spot associated with rhopalioid in *M. auricula*. Abbreviations: am, arm; cl, calyx; dp, dark pigment spot; pe, peduncle; rh, rhopalioid; st, secondary tentacles. Scale bar: A, C, D = 0.5 cm; B = 0.25 cm; E = 0.1 cm. Photo credits: A–B, A.G.C.; C–E, courtesy of Maciej Mańko.

During the metamorphosis of a stauropolyp into an adult stauromedusa, the eight primary tentacles (four interradial and four perradial) can have four fates: (1) they disappear by resorption (e.g., *Lucernaria*, *Craterolophus*, and some *Calvadosia*); (2) they metamorphose into adhesive interradial and perradial rhopalioids (e.g., *Manania* and *Haliclystus*); (3) they remain as primary tentacles but with a modified shape (e.g., some *Calvadosia*); (4) they change their shape (filiform to capitate), migrate and cluster together with the secondary tentacles (e.g., *Stylocoronella*) (see Miranda et al., 2016a). The secondary tentacles appear between two primary tentacles (one perradial and one interradial), in adradial position, and progressively get united in clusters during arm formation (Wietrzykowski, 1912) (Fig. 2).

We recently hypothesized that rhopalioids are a synapomorphy of the family Haliclystidae, which includes the genera *Manania*, *Haliclystus*, *Depastromorpha*, *Depastrum*, and *Halimocyathus* (Miranda et al., 2016a; Fig. 1). The structure likely has a role in temporary substrate attachment, a hypothesis supported by stauromedusan behavior (Larson, 1988) and histology (Miranda et al., 2016b). The genera *Lucernaria* and *Lipkea* are closely related, and we hypothesized that *Stylocoronella* would fit this clade (Fig. 1; see also Kikinger & Salvini-Plawen, 1995) based on the morphology (e.g., presence of interradial longitudinal muscles in peduncle and absence of primary tentacles or rhopalioids in stauromedusa) (Miranda et al., 2016a). *Craterolophus* and *Calvadosia* belong to the suborder Amyostaurida as they are the only two genera without interradial longitudinal muscles in peduncle (Miranda et al., 2016a).

In a recent article, Picciani et al. (2018) proposed that eyes (defined as a “region made of photoreceptor cells adjacent to pigment cells”, irrespective of image formation, i.e., including all photoreceptive organs) originated at least eight times in Cnidaria, even in the absence of a central nervous system. Their study was mainly based on a review of the literature on the presence of eyes for adult medusae (their Table S1) in light of an extensive ribosomal and mitochondrial-based molecular phylogeny for Cnidaria. Their analyses covered all of the cnidarian classes (other than the unusual parasitic class Myxozoa), including Staurozoa (i.e., stalked jellyfishes, Fig. 2), which was characterized as lacking eyes (Picciani et al., 2018).

Picciani et al. (2018) considered eyes absent in Staurozoa based on a simple statement by Mayer (1910 p. 520: “eyes [...] are absent in Stauromedusae”). Nevertheless, Picciani et al. (2018) correctly included an exception for the genus *Stylocoronella*. Polyps of *Stylocoronella riedli* Salvini-Plawen 1966 and *Stylocoronella variabilis* Salvini-Plawen 1987 possess dark pigment spots on the oral side of the calyx, at the inner bases of the tentacles (Salvini-Plawen, 1966; Salvini-Plawen, 1987; Blumer et al., 1995; Kikinger & Salvini-Plawen, 1995). Based on histological studies, these pigment spots were interpreted as being ocelli (Salvini-Plawen, 1966). Although the polyps of *S. riedli* show no distinct reaction to light stimuli, ultrastructural results corroborated the hypothesis that these structures are light-sensitive organs (Blumer et al., 1995). The ocelli are composed of seven to nine monociliary sensory cells, that lie next to the tentacular mesoglea, and one to four pigment cells (Blumer et al., 1995). The monociliary sensory cells of *S. riedli* show all the features characteristic for photoreceptive cells, including the intraciliary structure, demonstrating that these cilia are immobile (see Blumer et al., 1995). The pigment cells enclose the photoreceptive cilia and have irregularly shaped pigment granules enveloped by a membrane (Blumer et al., 1995). The dark pigment visible in living animals is associated with these membranes (Blumer et al., 1995). In addition, the ocelli in *Stylocoronella* have characteristics (e.g., arrangement of microtubules of photoreceptive cilia and membranous elements of pigment granules) that were hypothesized as unique for *Stylocoronella* and unknown within other metazoans (Blumer et al., 1995; Martin, 2002). The dark pigment spots are, in a somewhat modified arrangement, retained in the medusa stage (Blumer et al., 1995; Kikinger & Salvini-Plawen, 1995). The stauromedusa stage of *S. riedli* has “numerous tiny pigment spots in the basal area of the capitate tentacles and four larger perradial ones at the subumbrellar margin”, which were also presumed to be ocelli (Kikinger & Salvini-Plawen, 1995).

However, there are no molecular sequences for *Stylocoronella* species, so the presence of these ocelli was disregarded in the analyses of eye origins within Cnidaria (Picciani et al., 2018). On the other hand, Picciani et al. (2018) included two species of *Manania*: *Manania gwilliami* Larson & Fautin 1989 and *Manania uchidai* (Naumov 1961) (their Table S1) in their phylogeny, and both possess dark pigment spots (=eyes, *sensu* Picciani et al., 2018) associated with rhopalioids (= anchors) in the stauromedusa stage (Naumov, 1961; Larson & Fautin, 1989). Therefore, Picciani et al. (2018) neglected literature on Staurozoa and our aim is to review it and discuss the evolutionary implications of correctly coding this character in Staurozoa.

### Survey methodology

For recent papers reviewing the global diversity and natural history of stalked jellyfishes (Miranda et al., 2018), and their systematics (Miranda et al., 2016a), we compiled and reviewed literature containing every original description (50), occurrence, and morphological description of staurozoan species to our knowledge (see Miranda et al., 2016a; Miranda et al., 2018, and respective online resources). For this study, we updated our list of any new additions to the literature and searched for any detail that could speak to the possibility of eye-like structures using the terms “black”, “dark”, “pigment”, “eye”, “spot” in English, French, and German in the accumulated literature on Staurozoa.

## Results

### The genus *Manania* Clark 1863 (family Haliclystidae)

Currently, the genus *Manania* comprises seven valid species. Virtually all of them have evidence of dark pigment spots in adult stauromedusae.

*Manania auricula* (Fabricius 1780) and *Manania hexaradiata* (Broch 1907)

There is not a clear mention of dark pigment spots associated with rhopalioids for these two species in the literature. However, see Figs. 2C–2E showing a specimen identified as *M. auricula* from Svalbard with dark pigment spots associated with rhopalioids. Additionally, there are doubts regarding the validity of *M. hexaradiata*. Mayer (1910) considered *M. distincta* (see below) closely allied to *M. hexaradiata*. Uchida (1929) proposed that *M. hexaradiata* seemed a “young specimen of a medusa closely allied to, if not identical with” *M. distincta*.

*Manania distincta* (Kishinouye 1910)

The first unequivocal mention on the dark pigment spots in *Manania* in the literature was provided by Kishinouye (1910) in his description of *M. distincta*: “the eight primary tentacles are transformed into small, cylindrical bodies. They are erect, hollow inside, and not adhesive. They are black at base and along the axial median line. They serve probably as a sensory organ standing in relation to light”.

*Manania atlantica* (Berrill 1962)

Berrill (1962) mentioned that, at the base of each rhopalioid of *M. atlantica*, there is a “small spherical ocellus, apparently only a pigment spot”.

*Manania uchidai* (Naumov 1961)

Different records in the literature of *M. distincta* are actually *M. uchidai* (Figs. 2A–2B), as proposed by Naumov (1961). Uchida & Hanaoka (1933) mentioned that at the axial base of the rhopalioids of *M. uchidai* (as *M. distincta*) “there exists a blackly pigmented spot which probably serves as an organ standing in relation to light” and that “on the axial side of these tentacles [= rhopalioids] there is a sensory organ for light which is blackly pigmented and composed of exceedingly narrow cells arranged in a row”. Hanaoka (1935) added that “on the axial side of primary tentacles [=rhopalioids]” of *M. uchidai* (as *M. distincta*) “there is a sensory organ for light, which is blackly pigmented”. Uchida (1929), who also misidentified *M. uchidai* as *M. distincta*, highlighted that “in my specimen the pigment has probably faded away on account of preservation or has not yet appeared owing to its being young”. Later, Hirano (1986) observed that “the axial base of each primary tentacle (=rhopalioids)” was “provided with a black spot”.

*Manania gwilliami* Larson & Fautin 1989

Larson & Fautin (1989) described that each rhopalioid of *M. gwilliami* has a “small, dark pigment spot near margin”.

*Manania handi* Larson & Fautin 1989

In this unpublished thesis, Gwilliam (1956) described the species *Manania prasinus*, mentioning that “the adaxial side of the primary tentacles [= rhopalioids] bears a dark pigment fleck”. Later, the species was formally described by Larson & Fautin (1989) as *M. handi*, with a “dark spot on adaxial side of each primary tentacle [= rhopalioids] near margin”.

Recently, Westlake & Page (2017) showed that the pigment spot at the base of the rhopalioids of *M. handi* is associated with a greatly increased “concentration of FMRFamide-IR neuronal cell bodies”.

### The genus *Haliclystus* Clark 1863 (family Haliclystidae)

*Haliclystus* is the most diverse genus within Staurozoa, with 13 valid species (Miranda et al., 2018), but the presence of dark pigment spots in stauromedusae of this genus is more elusive. Naumov (1961) mentioned that the rhopalioids of *Haliclystus* are sometimes supplied with a pigmented eyespot. Gwilliam (1956), while describing *Haliclystus* species, observed that “there are no conspicuous pigment stripes other than the dark pigmented band on the subumbrellar side of the anchor [= rhopalioid] peduncle”.

More specifically, Clark (1878; see also his Fig. 27 and 32) observed, for *Haliclystus auricula* Clark 1863, “dark patch of color so noticeable at the proximal bases of tentacular groups, and which remind one of eye-spots” and dark spots associated with rhopalioids. Then, Clark (1878) clearly inferred the existence of a nervous system in *H. auricula* based on the presence of “eye-spots” found in rhopalioids: “We speak of these eye-spots because they occupy a position at the proximal side of the base of the anchors homologous with that in which a more highly developed and even well defined optical apparatus is to be found in other Acalephae. In our Lucernarian it amounts to a mere accumulation of pigment, in unusual quantity, in a small circle, among the interstices of the prismatic cells of a specially thickened wall […]. The boss-like protuberance of the wall at these spots, conjoined with the conspicuous coloring matter imbedded in it down to half its depth, give it strong claims to some special functional status, or to a typical representation of what finds its full development in other Acalephs. The accumulation of pigment matter at any point concentrates light there rather than any other force capable of being taken note of by a nervous centre. Neither odor nor sound would be affected by it, nor does it seem possible that taste could be seated at a point so distant from the digestive system. That it is after all a mere foreshadowing, or a mimetism, of a more efficient organ of vision becomes strongly probable when we learn that these spots lose their distinctness, or disappear altogether, by the time the animal measures one-half an inch across the umbrella. When the latter is about one-fifth of an inch across […] the spots have attained to their greatest definiteness, and from that period onward they gradually become obliterated; not so much, though, by fading out as by the increase of pigment all around them, until they lose their distinctness for want of contrast”. Clark (1878) added that “they are then probably to be set down rudimentary oculiferous tentacles situated within the line along which the anchors are disposed. Now in all Acalephae the eye, so called, stands in close proximity to the margin of the umbrella”. In addition, Clark (1878) mentioned that “we find it [pigment matter] holding exactly the same relation to the prismatic cells […], i.e., forming a dark casing or envelope about them, as the pigment does to the facets of the eyes of Articulata” and concluded “we have all that can be brought forward in favor of their functional characters as elements of an optical apparatus”. Therefore, it seems that the dark pigment spots associated with rhopalioids in *Haliclystus* disappear during the development, but their rhopalioids might still have knots of FMRFamide- immunoreactive neurons in adult stauromedusae (Westlake & Page, 2017).

In addition, but with less precision, Ling (1939) mentioned that *Haliclystus inabai* (Kishinouye 1893) has “anchors [= rhopalioids] brown with brown spot in center”.

### The genus *Stylocoronella* Salvini-Plawen 1966 (family Lucernariidae)

Dark pigment spots in the two species of *Stylocoronella* have been examined in detail (Salvini-Plawen, 1966; Salvini-Plawen, 1987; Blumer et al., 1995; Kikinger & Salvini-Plawen, 1995) in both stauropolyp and stauromedusa stages. Polyps of *S. riedli* and *S. variabilis* possess dark pigment spots on the oral side of the calyx, at the inner bases of the tentacles (Salvini-Plawen, 1966; Salvini-Plawen, 1987; Blumer et al., 1995; Kikinger & Salvini-Plawen, 1995). The youngest polyp observed of *S. riedli* had eight primary tentacles and already possessed eight pigment spots (Kikinger & Salvini-Plawen, 1995). The stauropolyp has up to 24 pigment-spot ocelli (at 24-tentacle stage), composed of monociliated sensory cells and pigment cells (Blumer et al., 1995; see also Martin, 2002). The cilia associated with the sensory cells (photoreceptive cilia) have a unique axonemal pattern, with a third central microtubule at a certain point (9 × 1 + 3 arrangement) and a balloon-like swelling of the distal portion of the cilium, with scattered microtubules in this area (Blumer et al., 1995; see also Martin, 2002). The stauromedusa stage of *S. riedli* has “numerous tiny pigment spots in the basal area of the capitate tentacles and four larger perradial ones at the subumbrellar margin”, which were presumed to be ocelli (Kikinger & Salvini-Plawen, 1995). The stauromedusa stage of *S. variabilis* does not have four perradial pigment spots (Kikinger & Salvini-Plawen, 1995).

### The genus *Calvadosia* Clark 1863 (family Kishinouyeidae)

The genus *Calvadosia* is the second most diverse in Staurozoa, with 11 valid species (Miranda et al., 2018). However, we found few mentions in the literature that could indicate (with imprecision) the presence of dark pigment spots in this genus. Kishinouye (1902) observed that the primary tentacles of *Calvadosia nagatensis* (Oka 1897) are absent and “in place of them we see a dark pigment for each”. Ling (1937) described that a “semi-triangular purplish area is seen in each of the eight marginal notches in close contact with the primary tentacles” for *Calvadosia cruciformis* (Okubo 1917) and a “semi-triangular purplish streak present at bottom of every marginal notch” for *Calvadosia tsingtaoensis* (Ling 1937), that faded away gradually after specimens are preserved.

## Conclusions

Based on our review, all dark pigment spots in staurozoans are associated with the primary tentacles, the region where they used to be, e.g., *Stylocoronella* and *Calvadosia,* or with the rhopalioids that are derived from primary tentacles (e.g., *Manania* and *Haliclystus*). Because the rhopalia of medusae of Cubozoa and Scyphozoa are also derived via metamorphosis of primary tentacles of cubopolyps and scyphopolyps, respectively, rhopalioids and rhopalia are hypothetically homologous (Thiel, 1966). However, whereas the rhopalia is clearly a sensory structure (reviewed in Katsuki & Greenspan, 2013), an adhesive rather than sensorial function thought to be associated with the benthic habit of staurozoans is often attributed to the rhopalioids of stauromedusae (Larson, 1988; Miranda et al., 2016b; Miranda et al., 2018).

Dark pigment spots associated with the eight rhopalioids have been observed in virtually all species of *Manania* and in at least some species of *Haliclystus*. We found no records of dark pigment spots for the other genera with rhopalioids, *Depastromorpha*, *Depastrum*, and *Halimocyathus* (Miranda et al., 2016a), in the literature, but this information could be overlooked, since the dark spots disappear after preservation (Uchida, 1929) and their presence can vary during development (Clark, 1878).

Unlike the pigment spots in polyps of *Stylocoronella* (Blumer et al., 1995), the ultrastructure of dark pigment spots in stauromedusae of *Manania* and *Haliclystus* have never been analyzed, potentially raising doubt about a sensorial function. However, recent evidence (Westlake & Page, 2017) supports the idea of photo reception by these organs in both genera. Westlake & Page (2017) analyzed the neuromuscular morphology of two stauromedusae, *Manania handi* (Larson & Fautin 1989) and *Haliclystus “sanjuanensis”* (*nomen nudum*), using whole mount immunohistochemistry with antibodies against FMRFamide and *α*-tubulin to label neurons. Comparative observations on cnidarians indicate that photoreceptive organs are consistently associated with the expression of these markers (see Westlake & Page, 2017), although peptides of the RFamide family also occur in species or life stages of cnidarians that do not have ocelli or known structures of photoreception (Plickert & Schneider, 2004). Interestingly, the “transformed primary tentacles” of *M. handi* had a greatly increased “concentration of FMRFamide-immunoreactive neurons at their base” associated with the dark pigment spots, which the authors concluded to be “consistent with their homology with rhopalia” of cubomedusae and scyphomedusae (Westlake & Page, 2017). Besides, a similar, but less pronounced, knot of FMRFamide- immunoreactive neurons is present at the base of the rhopalioids of *H. “sanjuanensis”*, although the species lacks a pigment spot in this area (although, as we pointed out, there are changes during development in *Haliclystus*). This result might be associated with the observed expression of opsin genes (the functional visual pigments in vertebrate and most invertebrate photoreceptors; Martin, 2002) in species of *Haliclystus* (Picciani et al., 2018), but raises interesting questions about the expression of medusozoan opsin genes reported by Picciani et al. (2018) in staurozoan species whose adult stauromedusae apparently lack both rhopalioids and dark pigment spots, such as *Lucernaria quadricornis* Müller 1776 and *Craterolophus convolvulus* (Johnston 1835). For example, What is the diversity of opsin genes across Staurozoa, and how have these genes evolved? Where and when are different opsin genes expressed across staurozoan life cycles and body regions? Are there mechanisms of extraocular photosensitivity in Staurozoa (see Martin, 2002)?

Although the dark pigment spots in *Manania* species were often associated with hypotheses of light perception, Westlake & Page (2017) mentioned that *M. handi* showed no immediate response to changes in light intensity, and hypothesized that “light sensitive neurons in staurozoans may detect light to trigger synchronous spawning, rather than to modify immediate behavior” (Westlake & Page, 2017). However, they highlighted that many stauromedusae spawn in response to light (see Otto, 1976; Otto, 1978; Miranda et al., 2018) despite having no obvious pigment spots (e.g., species of *Haliclystus*), which could be interpreted as a counterargument for this hypothesis (see examples of extraocular photosensitivity associated with cnidarian spawning in Martin, 2002). Besides, polyps of *Stylocoronella* also have ocelli, show no distinct reaction to light stimuli (Blumer et al., 1995) and do not spawn (as gonads are only fully developed in the stauromedusa stage; Kikinger & Salvini-Plawen, 1995). Therefore, although the ultrastructure of ocelli are compatible with light perception, at least in polyps of *S. riedli* (Blumer et al., 1995), the complete understanding of their function is a challenge and further studies that specifically address photosensitive behavior are necessary.

**Figure 3:**
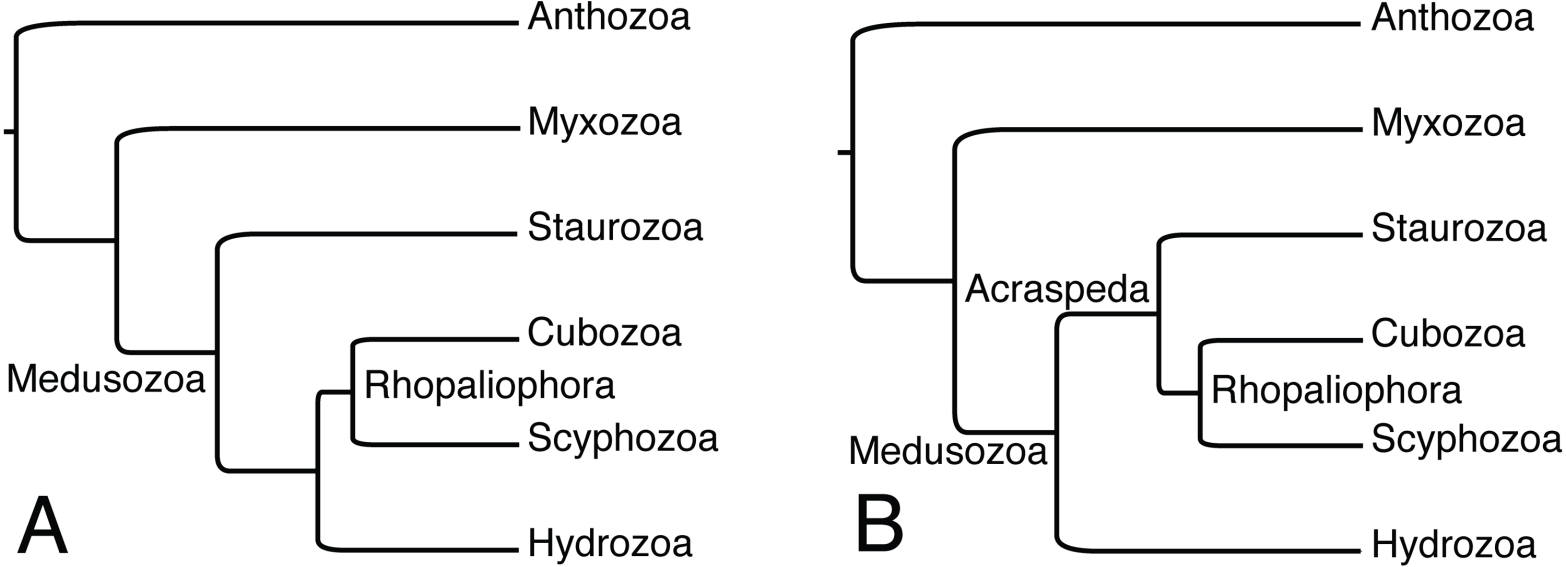
Alternative phylogenetic hypotheses for the placement of Staurozoa within Medusozoa. (A) Based on analyses of ribosomal and mitochondrial markers of Picciani et al. (2018). (B) Based on phylogenomic analyses of Kayal et al. (2018). Myxozoa has been added to (A) for comparability, but this group has generally not been treated in analyses of ribosomal and mitochondrial markers data due to highly accelerated rates of evolution.

The presence of dark pigment spots (or ocelli) in Staurozoa suggests that rhopalioids in stauromedusae can have both adhesive (see Miranda et al., 2016b) and sensorial functions. The reasons related to the wide occurrence of these structures in the stauromedusa stage of species of *Manania* needs further investigation. The homology of dark pigment spots in Staurozoa is questionable, since the genera *Manania*, *Haliclystus*, *Stylocoronella*, and (possibly?) *Calvadosia* do not form a monophyletic group (Miranda et al., 2016a; Fig. 1). In this context, ultrastructural studies on the dark pigment spots of different genera, especially *Manania*, and at different stages (i.e., polyp and medusa) should also be encouraged as it could provide relevant data on the evolution of this character in Staurozoa. However, in all these genera, the dark pigment spots of stauromedusae were associated with primary tentacles, their region (perradial/interradial) or structures derived from them. Therefore, based on our review and on the phylogenetic topology obtained by Picciani et al. (2018), in which Staurozoa formed a sister group relationship to the remaining medusozoans (Fig. 3A), the presence of these structures in Staurozoa could indicate at least a ninth independent origin of eyes in Cnidaria.

Alternatively, the observations reviewed here highlighting eyes associated with structures derived from primary tentacles (rhopalioids/rhopalia) might indicate shared ancestry of eyes of medusae across Cubozoa, Scyphozoa, and Staurozoa, which were recently shown to form the clade Acraspeda based on phylogenomic data (Kayal et al., 2018; Fig. 3B). The Acraspeda hypothesis linking Staurozoa with Cubozoa and Scyphozoa goes back to the late 1880’s (Haeckel, 1880; Claus, 1883) and was also suggested by a cladistic analysis based on morphology and life history characteristics (Marques & Collins, 2004). This explicit phylogenetic analysis supported the assertion of Thiel (1966) that rhopalia and rhopalioids, both derived from primary polyp tentacles, are shared across Acraspeda due to common ancestry. That these apparently homologous structures are also always the position of eyes across Acraspeda raises the possibility that light sensitivity has specific components that are shared across the group due to common ancestry. On the other hand, the strength of the Picciani et al. (2018) analysis was that it looked at species level observations of eyes, and while eyes are ubiquitous across Cubozoa, documented eyes are less than universal in both staurozoans and scyphozoans, supporting the idea that eyes may have evolved independently several times within Acraspeda. We support that eyes in hydromedusae have an independent origin from those in the medusae of Acraspeda, as sense organs in the former are not associated with metamorphosis of primary polyp tentacles (Thiel, 1966; Salvini-Plawen, 1987; Marques & Collins, 2004; Picciani et al., 2018). In short, correctly coding this character in Staurozoa, a lineage of benthic medusae, has profound consequences for understanding the evolution of eyes and nervous systems in Cnidaria and studies disregarding the presence of staurozoan ocelli should be reassessed.

## Additional Information and Declarations

### Data Availability

The following information was supplied regarding data availability: The research in this article did not generate any data or code as it is a literature review.
